# Maxillary Sinus Augmentation Using Autologous Platelet Concentrates (Platelet-Rich Plasma, Platelet-Rich Fibrin, and Concentrated Growth Factor) Combined with Bone Graft: A Systematic Review

**DOI:** 10.3390/cells12131797

**Published:** 2023-07-06

**Authors:** Giuseppina Malcangi, Assunta Patano, Giulia Palmieri, Chiara Di Pede, Giulia Latini, Alessio Danilo Inchingolo, Denisa Hazballa, Elisabetta de Ruvo, Grazia Garofoli, Francesco Inchingolo, Gianna Dipalma, Elio Minetti, Angelo Michele Inchingolo

**Affiliations:** 1 Interdisciplinary Department of Medicine, University of Bari “Aldo Moro”, 70124 Bari, Italy; giuseppinamalcangi@libero.it (G.M.); assuntapatano@gmail.com (A.P.); giuliapalmieri13@gmail.com (G.P.); c.dipede1@studenti.uniba.it (C.D.P.); dr.giulialatini@gmail.com (G.L.); ad.inchingolo@libero.it (A.D.I.); denisahazballa@gmail.com (D.H.); studio.deruvo@libero.it (E.d.R.); graziagarofoli.g@libero.it (G.G.); angeloinchingolo@gmail.com (A.M.I.); 2 Department of Biomedical, Surgical, Dental Science, University of Milan, 20161 Milan, Italy; elio.minetti@gmail.com

**Keywords:** PRP, PRF, CGF, Sinus Lift (S.L.), oral surgery, growth factor

## Abstract

Background: The current review aims to provide an overview of the most recent research on the potentials of concentrated growth factors used in the maxillary sinus lift technique. Materials and methods: “PRP”, “PRF”, “L-PRF”, “CGF”, “oral surgery”, “sticky bone”, “sinus lift” were the search terms utilized in the databases Scopus, Web of Science, and Pubmed, with the Boolean operator “AND” and “OR”. Results: Of these 1534 studies, 22 publications were included for this review. Discussion: The autologous growth factors released from platelet concentrates can help to promote bone remodeling and cell proliferation, and the application of platelet concentrates appears to reduce the amount of autologous bone required during regenerative surgery. Many authors agree that growth factors considerably enhance early vascularization in bone grafts and have a significantly positive pro-angiogenic influence in vivo when combined with alloplastic and xenogeneic materials, reducing inflammation and postoperative pain and stimulating the regeneration of injured tissues and accelerating their healing. Conclusions: Even if further studies are still needed, the use of autologous platelet concentrates can improve clinical results where a large elevation of the sinus is needed by improving bone height, thickness and vascularization of surgical sites, and post-operative healing.

## 1. Introduction

**Figure 1:**
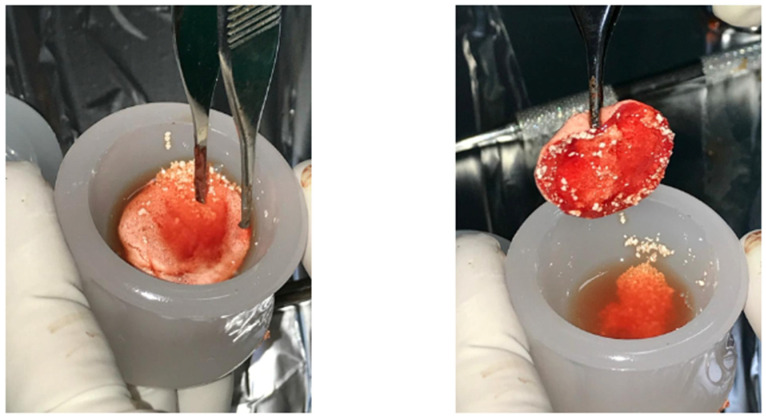
Sticky bone: A compact and plastic compound, evolution of the regenerative technique with CGF.

The balance of bone resorption and bone creation is critical for the preservation and regeneration of alveolar bone and supporting structures surrounding teeth and dental implants [1]. Tissue regeneration in the oral cavity is influenced by a variety of cell types, signaling systems, and matrix interactions [1,2]. Severe bone defects in the areas where the implant is to be placed might restrict the surgery; as a result, numerous bone regeneration techniques have been designed [3]. Maxillary sinus floor elevation (MSFE) is one of these procedures meant to increase bone volume in the atrophic posterior maxilla [4,5]. MSFE aims to increase bone height in the posterior maxilla by raising the Schneiderian membrane and inserting graft material into the surgically generated gap in the maxillary sinus floor [1]. Clinical trials of MSFE and other bone grafting methods, including biomaterial grafting, have been conducted to enable more predictable and strategic implant-supported prostheses [6]. Because of their osteoinductive and osteoconductive qualities, as well as their immunogenic compatibility, autogenous bone transplants have long been employed [3]. However, there are disadvantages to using autogenous bone transplants, such as donor site morbidity, insufficient quantity, and bone resorption in patients after a long healing period [7]. Although they provide the most biocompatible option, the disadvantages of autogenous grafts have driven the search for alternatives [8]. In current dentistry, the use of platelet-rich products derived from the patient’s own blood appears to be a favored therapeutic option [9]. Platelets serve as reservoirs for growth factors and cytokines that aid in bone and soft tissue regeneration during wound healing [10]. Platelets, when activated, establish a network in the fibrin matrix and release growth factors that drive the tissue-healing mechanism and, as a result, regeneration [4]. Platelets contain secretory granules that are rich in growth factors: vascular endothelial growth factor (VEGF), transforming growth factor-b1 (TGF-b1), platelet-derived growth factor (PDGF), epidermal growth factor (EGF), hepatocyte growth factor (HGF), fibroblast growth factor (FGF), insulin-like growth factor (IGF), etc. [10,11]. The main generations of APCs are platelet-rich plasma (PRP), platelet-rich fibrin (PRF) and concentrated growth factor (CGF) [4]. PRP, the first generation of platelet concentrates (PCs), is a plasma concentrate high in platelets that may be produced by centrifuging the patient’s venous blood and then using it as a bone-grafting material [12,13]. Because of the limits of PRP due to its anticoagulant composition, additional research by Joseph Choukroun in the early 2000s focused on generating a second-generation platelet concentrate free of anticoagulant factors [14]. The second generation of platelet concentrates, platelet-rich fibrin (PRF), has the same qualities as PRP but with the added benefit of osteogenicity [14]. The presence of additives in PRF is not required because of the presence of fibrinogen, which is converted to fibrin under the effect of physiologically accessible thrombin; this minimizes the risk of postoperative complications [9]. Concentrated growth factor (CGF), first used by Sacco in 2006, is a new platelet concentrate product that has demonstrated promising outcomes in soft tissue stimulation and acceleration as well as bone healing and creation [15,16,17,18]. CGF concentrates CD34+ stem cells and several growth factors in a small quantity of plasma [19]. CGFs are produced by centrifuging blood samples at alternate and regulated speeds in a specially designed centrifuge (Medifuge, Silfradentsrl, Italy) [20]. Different centrifugation rates allow for the separation of a fibrin matrix that is rich in growth factors and is significantly bigger and denser than PRP or PRF [20]. Sticky bone is an evolution of the regenerative technique with CGF. The sticky bone is obtained by mixing the centrifuged liquid of the test tube with a white smooth-walled cap that gels after a few minutes with the bone particulate of various kinds composed of mixed granulometry (calcium triphosphate, BiOss, dentin deriving from toot transformer) [21]. A compact and plastic compound is obtained that is easy to insert into the site to be regenerated. By then also combining the chopped fibrin clot before the gelling of the preparation, it is possible to obtain a compound richer in growth factors and therefore with greater regenerative potential [22] (Figure 1). The current review aims to provide an overview of the most recent research on the therapeutic and experimental potentials of autologous platelet concentrates (PRP, PRF and CGF) combined with bone grafts in maxillary sinus augmentation.

## 2. Materials and Methods

### 2.1. Protocol and Registration

This review was conducted in accordance with the standards of the Preferred Reporting Items for Systematic Reviews and Meta-analysis (PRISMA) [23].

### 2.2. Search Processing

PRP, PRF, CGF, ORAL SURGERY, STICKY BONE, SINUS LIFT were the search terms utilized in the databases (Scopus, Web of Science, and Pubmed) to select the papers under evaluation, with the Boolean operator “AND”.

The search was restricted to only items released in English during the previous ten years (2010–2023) (Table 1).

### 2.3. Eligibility Criteria

The reviewers, who worked in pairs, chose works that satisfied the following criteria for inclusion: (1) human subjects-only research; (2) clinical studies or case reports.

Exclusion criteria were (1) in vitro studies, (2) animal studies, (3) systematic reviews, narrative reviews, and meta-analyses.

The review was conducted using the PICO criteria:

Population: adults, both male and female who needed maxillary sinus lift;

Intervention: growth factors used in the maxillary sinus lift technique;

Comparison: maxillary sinus lift technique without growth factors;

Outcome: effectiveness in bone regeneration.

### 2.4. Data Processing

The screening procedure, which was carried out by reading the article titles and abstracts chosen in the earlier identification step, allowed for the exclusion of any publications that varied from the themes looked at. The complete text of publications that had been determined to match the predetermined inclusion criteria was then read. Reviewer disagreements on the choice of the article were discussed and settled.

## 3. Results

**Figure 2:**
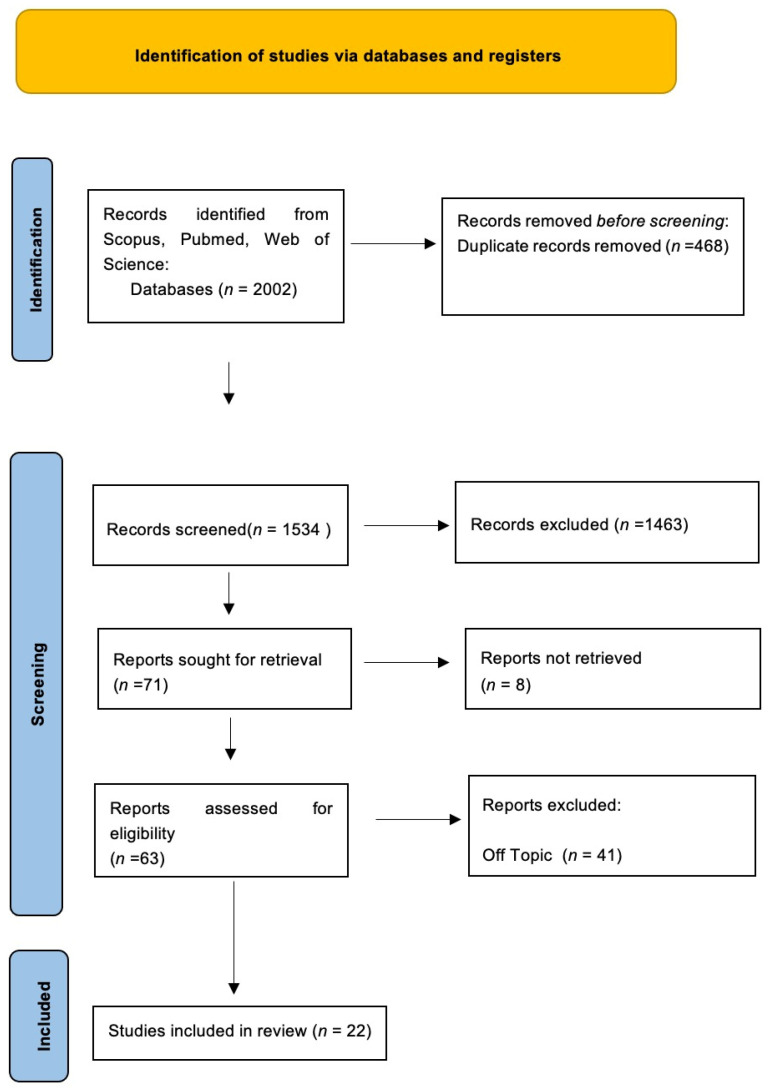
PRISMA flowchart.

Keyword searches in the Web of Science (307), Scopus (362) and Pubmed (1333) databases yielded a total of 2002 articles. The subsequent elimination of duplicates (468) resulted in the inclusion of 1534 articles. Of these 1534 studies, 1512 were excluded because they deviated from the previously defined inclusion criteria. The screening phase ended with selecting 22 publications for this work (Figure 2). The results of each study were reported in Table 2.

## 4. Discussion

### 4.1. Different Platelet Derivates

Based on different centrifugation parameters, platelet concentrates are classified into PRP, PRF and CGF [42,43].

PRP is a rich source of growth factors and platelets, and it is found in low-volume plasma. PRP includes FGF, TGF-β, IGF, PDGF-like growth factors and cell adhesion molecules such as vitronectin, fibrin and fibronectin. Because of this content, PRP accelerates wound healing [44].

Venous blood is drawn, and an anticoagulant agent is mixed in to prevent the blood from clotting. The mixture is centrifuged at 2400 rpm for 10 min. At the end of the first centrifugation, the blood in the tube is divided into two parts (upper part yellow plasma, lower part erythrocytes accumulate). The whole mixture, using the cannulation technique, is transferred to a second tube and subjected to a second centrifugation at 3600 rpm for 15 min to collect the platelet fraction at the bottom of the tube. What you get is PRP to be used for the surgical procedure [44].

Because of the limitations of PRP arising from its anticoagulant content, further studies by Joseph Choukroun in the early 2000s focused on the development of a second-generation platelet concentrate without the use of anticoagulant factors [45].

In this way, it was observed for the first time that in a single centrifugation cycle at 2700 rpm (750 g), a platelet concentration was collected that did not carry clotting factors to the top of the centrifuge tubes. This formulation is called PRF [46,47].

PRF is a second-generation platelet product that enables the formation of growth factors and platelet-rich membranes. PRF also contains leukocytes (WBCs) within the fibrin matrix (L-PRF) [46,48].

Peripheral venous blood is collected and centrifuged in glass-lined plastic tubes free of anticoagulants at 2700 rpm for 12 min or 3000 rpm for 10 min. Since there is no anticoagulant in PRF, coagulation begins when the blood is collected in the tube [8]. After centrifugation, a layer of cell-free plasma is formed at the top, a layer at the base rich in erythrocytes, and an intermediate layer of PRF clot. The PRF clot consists of a strong fibrin matrix in which platelets and leukocytes are concentrated [46,47].

PRF contains mainly platelets, fibrin, platelet growth factors, cytokines, leukocytes, circulating stem cells, monocytes, T and B lymphocytes, and neutrophil granulocytes [49].

CGF is a leukocyte- and platelet-rich fibrin structure first used by Sacco in 2006 [50]. As in PRF, CGF is obtained by a single centrifugation method. Plastic tubes without anticoagulants lined with red-capped silica particles are required, and no exogenous substances need to be added in this process [50].

The blood is centrifuged at low and controlled speeds for 12 min at 2400–2700 rpm. The resulting clot is divided into three layers (the upper layer contains platelet-poor plasma; the middle layer includes polymerized dense fibrin blocks containing fibrin and CGF; and the lower layer contains erythrocytes). The upper and lower layers are discarded, and CGF is collected in the buffy coat layer [51].

In 2006, Sacco first developed CGFs [52]. CGFs have stiffer fibrin structures than PRP and PRF [53]. In addition, CGF is more effective in bone regeneration and breast augmentation as it promotes osteogenesis [53,54].

CGF contains growth factors, such as PDGF, TGF-β, VEGF, insulin-like growth factors, epidermal growth factor, fibroblast growth factor, bone morphogenic protein and CD34+ cells [55]. CGF play an important role in vascular maintenance, angiogenesis and neovascularization [56].

A study by Dai et al. evaluated the efficacy of CGFs combined with MC in GBR [19]. Patients in whom CGF+MC was used and patients with MC alone were compared. It was seen that all implants healed, and the CGF+MC group had less swelling and less pain. The complex of CGF and MC seems to be appropriate and efficient as a biomaterial for bone augmentation [19].

The double-blind study by Ghasemirad et al. evaluated the effect of CGF on bone healing in a maxillary sinus lift [29]. A bovine xenograft was applied on one side and CGF on the other side. Staining with alizarin red and hematoxylin-eosin showed that the percentage of bone formed in the CGF group was significantly higher than in the control group [29].

So, the percentage of newly formed bone in the CGF group was significantly higher than in the control group (xenograft) after 6 months [29].

Zhang et al. evaluate the influence of PRF on bone regeneration in a xenograft-associated sinus lift (deproteinized bovine bone) [41]. On histological examination, no statistically significant differences were found between patients treated with PRF and patients treated only with xenograft. In conclusion, the study showed no differences of the application of PRF associated with deproteinized bovine bone in sinus augmentation 6 months after surgery [41].

### 4.2. Different Bone Graft Materials Used in Combination with Platelet Derivates

The “gold standard” for bone tissue regeneration is an autograft taken from an adjacent site in the same patient [57]. This procedure has negative effects such as a second surgical procedure, unpredictable extent of resorption, and shortage of donor sites [58,59]. So, bone substitutes can be applied to avoid these disadvantages.

The maxillary sinus has been augmented using a variety of graft materials, including autograft, xenografts and allografts, each of which has advantages and disadvantages [60]. PRF has greater advantages than other graft materials since platelets are essential for the development and repair of soft tissue and bone [61,62].

PRP is a plasma concentrate high in platelets produced by centrifuged peripheral venous blood from the patient and used as a bone-grafting material. PDGF, TGF-β, and VEGF are three growth factors that are particularly abundant in PRP, and they have a potential range of cellular activities that includes cell differentiation, tissue healing, angiogenesis and increasing collagen formation [61,62]. This approach had already been applied in other fields of medicine such as in dermatology up until the late 1990s when Marx et al. discovered that the use of PRP in conjunction with autologous bone might result in a noticeably better outcome [63].

The following cellular processes are stimulated by PRP three days after grafting in the recipient site: proliferation of osteoblasts and fibroblast, neoangiogenesis, and stimulation of the mineralization of the newly created bone matrix [1,64].

In implantology, GBR operations frequently employ PRP to repair edentulous regions that need an increase in bone volume [61].

A study by Inchingolo et al. involved a cohort of 127 patients requiring a maxillary sinus lift. Half of the patients received PRP in combination with anorganic, organic or autogenous bone; the control group received only grafting material without PRP. In all cases, authors obtained successful results, but the test group with PRP showed a statistically significant enhancement in osseointegration in terms of primary stability and peri-implant bone quality evaluated in tomographic sections with a 3D software [1].

Another strategy is to insert the implants during the sinus lift to save time and prevent a second surgery; Inchingolo et al. assessed the efficacy of PRP with deproteinized bovine oss (Bio-OSS) and beta-tricalcium phosphate (SINT-Oss) for a sinus lift and simultaneous implant placement in patients with sinus pathology [31]. The PRP prepared with Choukroun’s technique was used in two different ways: a portion was blended with Bio-Oss and Sint-Oss; the remaining was modelled as a resistant fibrin membrane that could be transferred to the Schneiderian membrane, and the other portion was transferred to the material used before closing the lateral window created with the use of piezosurgery. The soft tissues around the implants in all patients showed no signs of tissue damage; the implants had optimal primary stability, and the density of the bone around the implants had increased. There was no unfavorable progression of the sinusitis. The authors concluded that the combination of PRF and Piezosurgery decreased the healing time, favored optimum bone regeneration, and allowed sinus membrane integrity to be preserved during surgical treatments [31].

Instead, Kempraj et al. compared the use of Choukroun’s PRP as a single-graft material to Xenograft (BIO-OSS) for a sinus lift [34]. The sample size was constituted by 22 interventions performed with the lateral window technique. Compared to the use of PRP alone, radiological results revealed an important rise in bone density and in bone height in the Bio-oss group. This could be caused by the sinus membrane’s collapse of the PRF plug due to the absence of a structure that support it, as also reported by Lundgren et al. [65].

Promising results were shown by Powell et al., who experimented with L-PRF using three different methods: in the first case, L-PRF was employed to support a maxillary hybrid denture by bilateral sinus augmentation. In the second patient, it emphasized the use of L-PRF associated with an elevation of the Schneiderian membrane. In the third patient’s implant placement after L-PRF/xenograft sinus augmentation, a histological examination was provided six months later [40]. Dental implants were successfully placed in every patient; in the second case, freeze-dried bone allograft offered around 4 mm of extra vertical height for implant insertion. Six months after sinus augmentation, histology from the third case showed that there was fresh, viable bone in contact with the xenograft [40].

Simonpieri et al. stated that literature findings regarding PRP’s efficacy could have been clearer because different PRPs were tried in several different combinations with varied bone materials [9].

**Figure 3:**
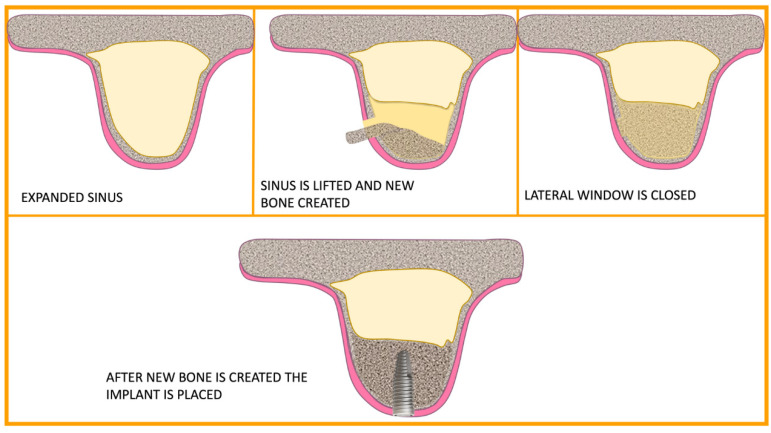
Timing of the sinus lift using lateral window technique.

**Figure 4:**
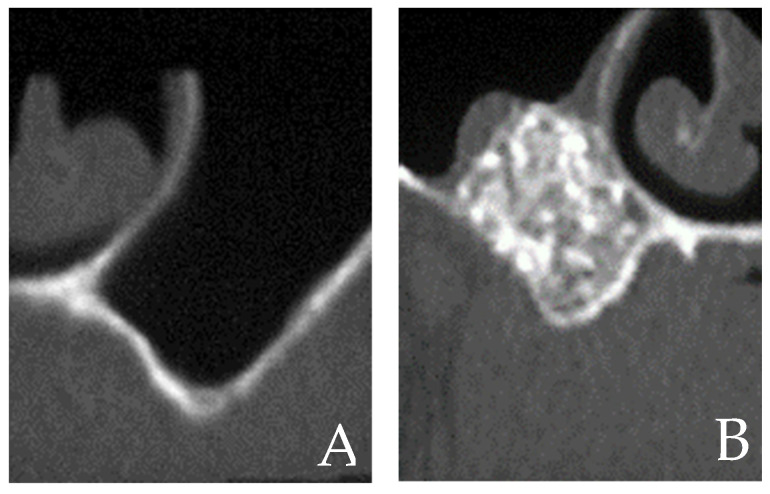
Section of a CBCT before (**A**) and after (**B**) the sinus lift.

Despite being an excellent way to manage bone graft material during the incision in the subsinus cavity, and despite having the potential to speed up bone healing, the degree of proof for this technique was still just marginal because the surgical treatment already had a very high success rate even without PRP [9]. Surgical methods and how PRP and bone grafts were combined varied between research, so Simonpieri et al. asserted that because of the many methodological variations, it could appear hard to draw broad conclusions from the diverse research present in literature [9] (Figure 3 and Figure 4).

### 4.3. Surgical Techniques for Sinus Augmentation Using Platelet Derivatives

**Figure 5:**
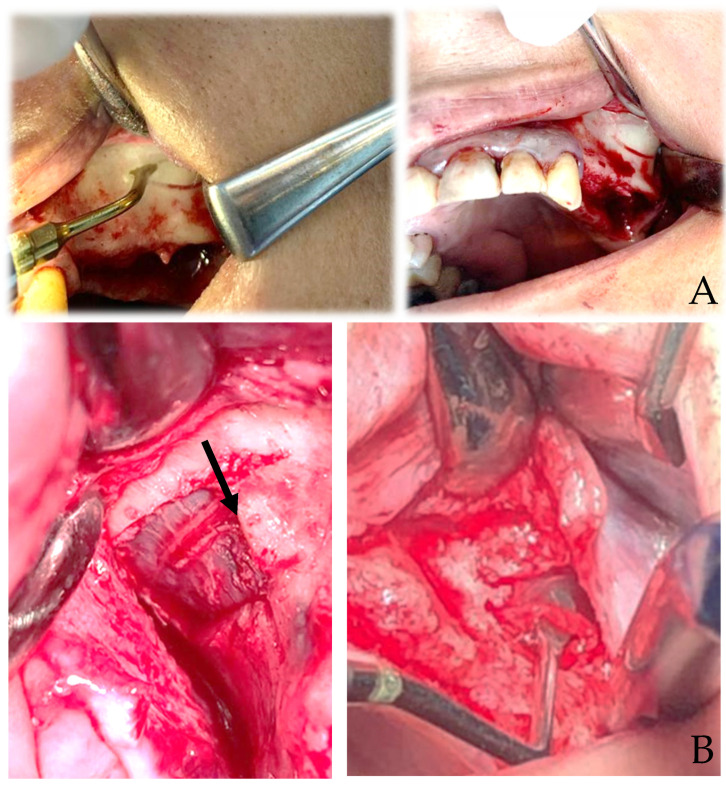
(**A**) Using piezo surgery to open the window of Tatum and protect the arteries and the Schneiderian membrane during the sinus lift graft (**B**) Views of the antral alveolar artery indicated by arrow.

Tatum, in 1976, modified the Caldwell–Luc technique and performed the first maxillary sinus lift procedure. Through the lateral window, the membrane of the sinus was dissected and elevated (Figure 5); in this case, autogenous bone was used as a bone substitute in the sinus, and the implant was placed after 6 months [66]. Boyne and James proposed the Caldwell–Luc sinus revision and the lateral window sinus floor elevation, and implant placement was performed in 3 months [66]. Since the first sinus floor elevation, numerous graft techniques and materials have been proposed.

In 1986, Tatum Jr. developed the transalveolar sinus floor elevation to minimize pain and suffering after surgery [67]. Summers modified this approach in 1994 [68].

The Summer’s osteotomy, which used no grafting material even in thin residual bone height, has been associated with developing the idea of limited grafting. The bone’s and the sinus membrane’s osteogenic potential is well-protected and effective in a closed space like an elevated sinus [69,70].

**Figure 6:**
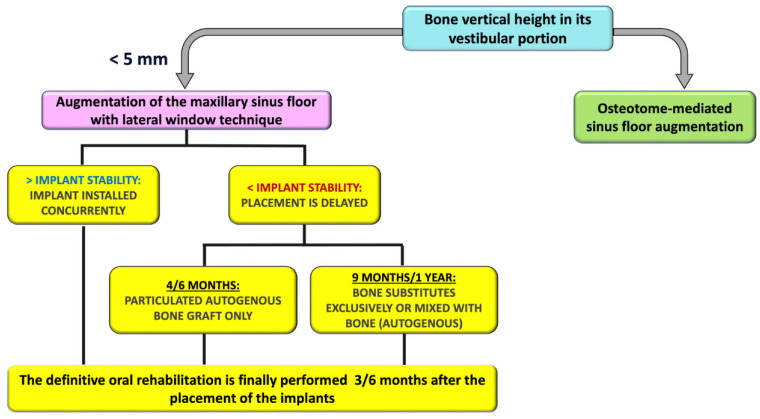
Surgical approach techniques for sinus lift and timing for implants.

Simonpieri et al. applied a sinus lift with the lateral window technique in 24 patients, and the follow-up time for these patients from the placement of the implants was 2–6 years (Table 2). The patients had a subantral augmentation category 4 (SA4) sinus morphology where the height of the crestal bone from the floor of the sinus is <5 mm. A PRF membrane was used for the Schneiderian membrane protection, and the implant served as “tent pegs” for the L-PRF-patched Schneiderian membranes. In this study, the height of the peri-implant crestal bone was consistent, and the floor level of the reconstructed sinus was always continuous with the apical edge of the implant [71] (Figure 6).

In fact, if the preservation of the Schneiderian membrane is achieved at the right height with the help of immediate implantation, this technique has given successful results [72]. But this technique does not accept tears of the sinus membrane and presents difficulties in filling the base of the sinus cavity with a blood clot [71]. Kaarthikeyan et al. concluded that PRF is an effective biomaterial when used alone for filling the maxillary sinus with an implant as a tentacle (Table 3), but perforation of the sinus membrane during the procedure may lead to unsatisfactory results [33].

Other authors have shown that the lateral approach can be performed in a full sinus lift only with whole blood and no other graft material [73,74]. Chitsazi et al. (Table 2) used only PRF as bone graft material for raising the maxillary sinus with an open window on one side and did not use graft material on the other side. Implants were placed in one session. This study (Table 3) stated that PRF may improve both the quantity and quality of bone resorption [27].

The fibrin network found in PRF has the tendency to develop a three-dimensional structure comparable to the place of insertion, promoting the healing process. A three-dimensional scaffold is created by the accumulation of fibrin monomers, creating a thin mesh of soft porous material that enables the quick cell colonization of the site and surrounding tissues [9,75,76].

Molemans et al. also conducted a study using only L-PRF as filler material in a maxillary sinus lift. Only in cases when the crestal height was <5 was the lateral window technique performed; in contrast, the preference was for the crestal technique (Table 2). The results showed that this biomaterial can be used in lateral sinus surgery with success. Implant failure was seen only in the crestal technique; it is possible that the membrane was perforated during the procedure [37].

Choudhary et al.’s goal was to assess the effects of simultaneous implant insertion with PRF and indirect sinus lift with hydraulic pressure when the average mean height at the beginning was 5.573 ± 0.66 mm (Table 3). The average pretreatment mean height significantly increased after surgery (Table 2). A six-month postoperative period saw an increase in the implant stability quotient [28]. These results were in line with earlier research that showed a considerable rise in residual alveolar ridge height after indirect sinus lift and concurrent PRF implant insertion [77,78].

During Summer’s osteotomy, the use of PRF membranes offered a good result as filling material. PRF servs as a cushion shock absorber during osteotomy and supports healing in case of a damaged Schneiderian membrane [79]. Huang et al. showed how the PRF membrane can be used to repair the perforation of Schneider’s membrane caused by the maxillary sinus lift procedure with the lateral window technique. The Schneiderian membrane’s perforation could be repaired by a PRF membrane [30]. The PRF membrane’s fibrin and platelet contents may both play a role in this impact [65,80,81].

Independently of whether a procedure was 1-stage or 2-stage, Rosen et al., for the osteotomy sinus floor elevation, found that the success of implant placement was better when the ridge bone height was ≥5 mm [82]. Other authors had shown that when the bone crest is less than 5 mm, the failure rate increases [83]. However, Li [84] asserts that if primary stability has been attained, the osteotomy procedure can be applied even in residual ridges with heights of 3–4 mm. Krasny et al., in 26 patients with a residual bone height of 3–5 mm using the transalveolar sinus lift technique in two stages, successfully reconstructed the maxillary sinus [85].

Aoki et al. presented the results of histopathological analyses performed in two case reports wherein sinus elevation was conducted—in one case with a lateral window and with only PRF as bone filling material and placement of implants in two stages, and in the second case a crestal approach with PRF as a bone-filling material (Table 2). The residual bone height in both cases was <2.7 mm. The histopathological results showed that the presence of PRF in the sinus cavity induced the formation of new bone [25].

The Schneiderian membrane has a high potential for osteogenesis, which explains why the majority of graft materials result in bone development [86,87,88]. Without the use of graft material, a sinus floor elevation can still be performed with enough bone development and implant longevity [72,89,90]. However, in an animal study, Kim et al. shown that bone development is restricted when no material for grafts is used in sinus lift surgery [91]; also, Sul et al. asserted that without graft material, bone formation may be constrained and that the implant apex could get caught with the Schneiderian membrane [92]. But in the study by Simonpieri et al., it was thought that the presence of the PRF membrane does not allow the implant apex to be enmeshed with the sinus membrane [71].

**Table 3:** Results (from 6–12–18 months after surgery) of a comparison between platelet-derived and different graft materials used for bone regeneration in the sinus lift technique. RBH, residual bone height before surgery; MBL, marginal bone loss; TBH, total bone height.

Author	Groups	RBH (mm)	MBL (mm)	TBH (mm)	Implant Success
Chen et al., [88]	Group A, CGF with bone grafting; Group B, CGF without bone grafting	A—5.01 ± 0.64 B—5.23 ± 0.49	A—0.11 ± 0.02 B—0.10 ± 0.02		Implant success rate was 100% in the two groups after 24 months
Kaarthikeyan et al., 2019 [33]	Group A, PRF Group B, Blood only	A—0.420 ± 0.480 B—6.391 ± 0.807		A—11.154 ± 2.392 B—11.916 ± 1.213	No implant failed after 12 months
Merli et al., [36]	Group A, DBBM Group B, CGF	A—2.3 (0.8) B—3.0 (0.8)	A—0.04 (0.1) B—0.2 (0.2)	A—9.4 (1.1) B—9.7 (1.9)	No implant failed for the two groups after 12 months
Chitsazi et al., [27]	Group A, PRF Group B, none	A—5.85 ± 1.08 B—5.67 ± 1.03		A—10.71 ± 1.09 B—9.28 ± 1.28	No implant failed in both groups after 6 months
Lv et al., [35]	Group A, PESS Group B, LSFE	A—3.35 ± 0.79 B—2.92 ± 0.63	A—0.60 ± 0.25 B—0.69 ± 0.35	A—7.67 ± 1.29 B—10.32 ± 1.26	PESS—96.15% LSFE—100% after 3, 6, and 9 months
Choudhary et al. [28]	Indirect sinus lift with hydraulic pressure using PRF.	5.573 ± 0.66 to		9.603 ± 0.78	ISQ was 72.92 ± 2.71 after six months

Anitua et al. conducted a study of bilateral maxillary sinus elevation where one side was treated with bovine bone and plasma rich growth factor (PRGF) and the other side as a control group only with bovine bone (Table 2). The results showed that the side where PRGF was used with bovine bone created new bone faster and was denser and more compact than that of the control group; in addition, the side that was treated only with bovine bone was more inflamed compared to the side where PRGF was used. Patients indicated pain on the side where only bovine bone was placed [24].

Platelet products have been found to inhibit monocyte cytokine release and restrict inflammation [93]. Additionally, new findings imply that platelets initially block the release of interleukin-1 (IL-1) from activated macrophages. Broad implications for the description of a process by which platelet-rich products may operate as an anti-inflammatory agent could result from the first reduction of the inflammatory response [3].

According to Lv et al., the flapless endoscope-supported osteotome sinus floor elevation using sole (PRF) has a lower incidence of postoperative pain and edema than sinus elevation with lateral windows filled with bovine bone and is more bearable for patients (Table 2). But compared to the transcrestal approach with PRF alone (Table 3), the lateral window technique with bovine bone seems to offer more peri-implant bone height and density [35].

Rapone et al. observed over a period of 7 years the results obtained from the elevation of the maxillary sinus with the lateral window technique [3]. Patients were divided in two groups, and as grafting material, the natural porous fluorohydroxyapatite combined with PRF was used in one group, and bovine bone with autogenous bone (50:50) combined with PRP was used for the other group. For both groups, this study revealed predictable outcomes over time (Table 2). Compared to implanting an ungrafted maxillary, this method offers a better long-term prognosis and a greater survival rate [3,94].

Irdem et al. stated that over a four-month period, the combination of bovine bone and liquid PRF helped create new bone, although this effect was not statistically significant compared to bovine bone alone [32]. Nizam et al. (Table 2) also came to the conclusion that under histological and histomorphometric examination, the addition of L-PRF to particle DBBM did not increase the amount of regenerated bone or the degree to which the graft was integrated into the newly created bone [39].

**Figure 7:**
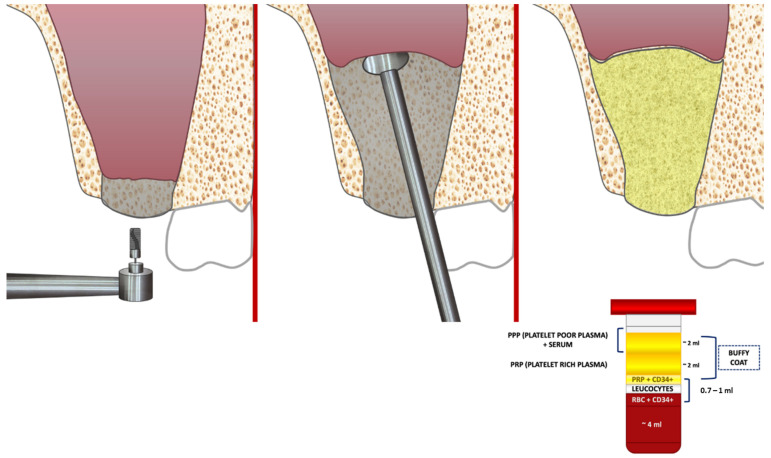
Summer’s technique using CGF graft for sinus lift.

Narang et al. [38], in a case report, used PRF with bone graft material to reach a height >10 mm in the maxillary sinus area when the patient had residual ridge heights of 1.49 mm and 1.47 mm. The technique followed was the modified Summer’s. The results showed that this method is successful in raising the maxillary sinus and placing the implants (Table 2). This procedure often only needs 3–4 months of recovery compared to other techniques, which typically need at least 6–9 months. One explanation would be the smaller access hole established in the sinus cavity. Blood flow is rarely affected by this approach. The main benefit of this technique, unlike the lateral window technique, is that the implant and bone grafts obtain most of their blood supply from buccal [38] (Figure 7).

Also according to Merli et al. (Table 2), lateral sinus floor elevation using CGFs as the only grafting material resulted in implant success rates and slight changes in bone level that were comparable to demineralized bovine bone grafting [36], Between the CGF and the DBBM groups, there was no statistically significant difference in marginal bone loss (Table 3).

In fact, CGF is considered a new generation of platelet products that have dense fibrin networks and a high concentration of GF and are important in cell proliferation [95]. The CD34+ cells have been discovered at both levels (CGF-RBC) and are entrapped in the CGF matrix in large numbers. Due to its promotion of osteogenic cell differentiation and proliferation, the CGF seems to have more promise for tissue regeneration. As a result, the CGF greatly boosts alkaline phosphatase (ALP) activity [52,96].

According to Chen et al., in patients with a residual bone height of 4–6 mm before surgery (Table 3), the osteotome sinus floor elevation with a CGF approach is safe and dependable, whether bone grafting is used or not. Individuals who had bone grafting experienced postoperative discomfort and pain compared to individuals who did not get bone grafting. For the two groups, there was no big difference in marginal bone loss [26].

According to some studies, the survival rate of implants inserted in augmented sinuses is not improved by autogenous bone alone [97,98,99,100]. The disappointing 82% implant success rate once autogenous block graft is employed was also highlighted in two of these reviews [98,99].

On the other hand, when xenograft was utilized instead of autogenous bone, two reviews found that 96% of 10,000 studied implants survived after sinus augmentation [97,98].

In the study developed by Forabosco et al., in the group using only xenograft, a 96.1% survival rate was recorded; in the group using a combination of CGF and xenograft, a 96.4% survival rate was recorded [101].

Chen et al., reported a 100% implant success rate in the two groups, which had either CGF with bone graft or CGF without bone graft [26]. Other studies that have used only CGF as graft material in maxillary sinus elevation [54] state that the bone level obtained and the success of the implants can be compared to that of a bovine bone graft [36].

## 5. Conclusions

In conclusion, there are different surgical methods for the treatment of peri- and pre-implant defects, from the use of zygomatic implants to the use of biomaterials capable of increasing and accelerating bone formation. Following tooth extraction, a process of bone resorption occurs, making it necessary to increase bone, especially with a view to implant-supported rehabilitation. MSFE has become a standard surgical procedure to solve the reduced levels of bone, allowing the positioning of dental implants. Different biomaterials have been proposed from synthetic products up to heterologous or autologous grafts for the purpose of bone conservation and regeneration.

Histological analyses reveal an enhanced vascularization and the early formation of new bone thanks to the use of growth factors. The significant positive pro-angiogenic influence of PRF combined with bone grafts favors regeneration processes, exploiting the body’s natural ability to repair injured bony tissue with new bone cells. The improved vascularization of the surgical site through neoangiogenesis promotes the healing of surgical wounds, and this is particularly advantageous, especially in surgical areas with reduced vascularization, such as in sinus lifts. Pre-treatment with PRP provides primary stability, improving in a statistically significant way implant–prosthetic rehabilitation. Comparing other bone substitutes, the sinus floor elevation performed with the use of CGFs alone showed implant survival and marginal bone level changes comparable to a demineralized bovine bone matrix.

Even if further studies are still needed, the use of CGF, PRF and PRP seems to have the ability to improve clinical results by improving the vascularization of surgical sites, and they can improve the post-operative quality of life of patients.

Platelet derivatives will certainly see further developments in the near future, which will above all improve costs, preparation time, and surgical efficiency, given that clinicians often complain of having minimal clinical advantages in the face of an expensive and complex procedure.

Furthermore, it will certainly be necessary to work towards making these procedures less operator-dependent, given that nowadays the effectiveness of these surgical procedures is very much linked to the surgical skills of the clinician, their knowledge of these materials and their ability to use them in the right way.

## Figures and Tables

**Table 1:** Database search indicators.

Article screening Strategy	Database: Scopus, Web of Science and Pubmed
	Keywords: A “PRP”; B “PRF”; C “L-PRF”; D “CGF”; E “ORAL SURGERY”; F “STICKY BONE”; G “SINUS LIFT”
	Boolean variable: “AND” and “OR”
	Timespan: 2010–2023
	Language: English

**Table 2:** Results table.

Authors (Year)	Type of the Study	Aim of the Study	Materials	Results
Anitua et al., 2012 [24]	A report of five cases	In five consecutive patients who had bilateral sinus lift augmentation, the prospective effects of (PRGF) technology were assessed.	-Five patients; -One side treated with bovine bone and PRGF, and the other only with bovine bone; -Lateral wall technique; -Two stage implants; -Five-month histomorphometrical analysis.	PRGF may play a role in lowering tissue inflammation during surgery, boosting the production of new bone, and encouraging the vascularization of bone tissues.
Aoki et al., 2016 [25]	Two cases reported	To describe histological result of PRF used as the only graft material for sinus augmentation.	-Two patients; -Patient A—PRF as sole graft material; -Osteotom crestal technique; -Simultaneous implant placement; -Patient B—PRF as sole graft material; -Lateral window technique’ -Two stage implant placement.	Patient A—implant fail. Histological analysis supported new bone growth in both situations.
Chen et al., 2021 [26]	A retrospective study	To examine the clinical effects of (OSFE) combined with (CGF) and simultaneous implant implantation with or without bone grafting.	-Forty-four patient; -Sixty implants; -Transalveolar technique with CGF combined or not combined with graft material; -Twenty-four-month follow-up.	OSFE with CGF approach is safe and dependable, whether bone grafting is used or not.
Chitsazi et al., 2018 [27]	Clinical trial	Following an open sinus lift treatment with and without PRF, an analysis of the height and density of the posterior of the maxillary bone	-Fourteen patients; -Lateral wall osteotomy; -Forty-one implant placements; -One side used PRF. The other side without graft material; -Follow-up at 6 months.	PRF may improve both the quantity and quality of bone resorption. No implant fail.
Choudhary et al., 2022 [28]	Clinical study	To evaluate indirect sinus lift with hydraulic pressure and simultaneous PRF implant implantation.	-Twenty-four patients; -Indirect sinus lift with hydraulic pressure; -Simultaneous PRF implant implantation; -Evaluation at six months.	The average pretreatment mean height significantly increased after surgery. After six-months, there was seen an increase in the implant stability quotient.
Dai et al., 2020 [19]	Retrospective study	The aim of the study was to evaluate the clinical efficacy of CGF combined with mineralized collagen (MC) in guided bone regeneration (GBR).	-Fifteen patients treated with CGF+MC (study group); -Fourteen patients treated with only MC (control group).	New bone growth and postoperative pain were both positively impacted by the use of CGF and MC. This indicates that the CGF-MC complex is a suitable and effective biomaterial for bone augmentation.
Ghasemirad et al., 2023 [29]	Randomized controlled trial	The aim of the study was to examine the effect of CGF on the healing of a maxillary sinus lift.	Nine patients undergoing maxillary sinus lift. Bovine xenograft was randomly applied to one side and CGF to the other side.	After 6 months, the proportion of newly produced bone in the CGF group was considerably higher than in the control (xenograft) group, according to alizarin red and hematoxylin-eosin staining procedures. The amount of leftover material in the control group compared to the intervention was noticeably higher.
Huang et al., 2016 [30]	Case report	To offer a quick and straightforward solution for Schneiderian membrane perforation repair.	-A 62-year-old man; -lateral wall protocol; -PRF to cover Schneiderian membrane perforation; -Synthetic bone graft and implant placement; -Thirty-month control.	The Schneiderian membrane’s perforation could be repaired by PRF.
Inchingolo et al., 2012 [1]	Prospective study	To demonstrate the efficiency of PRP as a grafting material in bone regeneration for dental implant.	-Sixty-three patients (study group): PRP+ autogenous or organic or anorganic bone; -Sixty-four patients (control group): bone graft without PRP.	The test group showed a statistically significant enhancement in osseointegration in terms of primary stability and peri-implant bone quality evaluated in tomographic sections with 3D software (Master 3D).
Inchingolo et al., 2015 [31]	Prospective study with 48 months of follow-up	To assess the results of maxillary sinus lift using PRF as a filling material in conjunction with the Bio-Oss and Sint-Oss and concurrent implant placement in patients with sinus pathology.	A total of 175 implants were placed after sinus lift using PRF in combination with deproteinized bovine oss (Bio-OSS) and beta tricalcium phosphate (SINT-Oss).	The soft tissues around the implants in all patients showed no signs of tissue damage, the implants had optimal primary stability, and the density of the bone around the implants had increased. There had been no unfavorable progression of the sinusitis.
Irdem et al., 2020 [32]	A Split-mouth, histomorphometric study	To determine if a liquid PRF-DBBM combination is successful at stimulating the growth of new bone during maxillary sinus augmentation.	-Seven patients; -Two groups, grafted with deproteinized bovine bone mineral (DBBM) with liquid PRF or DBBM alone; -Lateral wall protocol; -Two-stage implants; -Four months of histomorphometric evaluation.	The combination of DBBM and liquid PRF helped to build new bone, although this effect was not statistically significant.
Kaarthikeyan et al., [33]	A randomized controlled trial	To compare bone growth in the elevated maxillary sinus using an implant as a tent pole and PRF or blood clot alone as the only sinus-filling material.	-Seven patients; -Lateral bony window; -Implant used as a tent pole; -One side PRF, other side only blood; -Twelve-month follow-up.	As the only material to fill the sinuses, PRF might be a better option than a blood clot.
Kempraj et al., 2020 [34]	Radiological study	To compare the use of PRF as a single graft material to Xenograft (BIO-OSS) for sinus lift.	The sample size was constituted by 22 interventions of sinus lift with lateral window technique using Bio-oss (group 1) or PRF (group 2).	Compared to the use of PRP alone, radiological results revealed an important rise in bone density and in bone height in the Bio-oss group.
Lv et al., 2021 [35]	A randomized controlled trial	To assess the outcomes of simultaneous implant insertion and to compare outcomes between the PESS approach and lateral sinus floor elevation (LSFE).	-Forty patients; -PESS with PRF as the sole grafting material (transcrestal); -LSFE with deproteinized bovine bone matrix (lateral wall); -Simultaneous implant insertion; -Control 3, 6 and 18 months post-surgery;	PESS was more acceptable than LSFE and was linked to lower postoperative morbidity.
Merli et al., 2022 [36]	Controlled Clinical Trial	Analyzed the results of implants placed in the maxillary sinuses using a one-stage lateral technique and either (CGFs) or (DBBM).	-Twenty patient; -Lateral wall technique; -CGFs or DBBM as the sole grafting material; -Simultaneous implant placement; - Twelve-month follow-up.	No implant failed for the two groups. Between the CGF and DBBM groups, there was no statistically significant difference in marginal bone loss (MBL) change.
Molemans et al., 2019 [37]	Case series, single cohort prospective study	To evaluate the results of simultaneous implantation and SFE utilizing leukocyte- and platelet-rich fibrin (L-PRF) as the sole graft material.	-Twenty-six patients (28 SFE); -Six lateral sinus lift; -Twenty-two transalveolar sinus lifts; -L-PRF as a sole graft material; -L-PRF to protect the Schneiderian membrane; -Follow-up at the time of implantation and after 6 months.	L-PRF has proven to be a useful, safe, and affordable subsinus graft material, resulting in natural bone growth when used as the only graft material.
Narang et al., 2015 [38]	Case report	Suitable techniques and materials that can increase bone thickness by more than 10 mm using the osteotomy procedure and grafting materials.	-A 67-year-old female patient; - Summer’s osteotomes modified techniques; -PRF combined with bone graft material.	During the OSFE procedure and implantation, using PRF and bone graft material is a safe and efficient alternative.
Nizam et al., 2017 [39]	Randomized controlled clinical trial	To assess how (L-PRF) in conjunction with (DBBM) affects bone regeneration in maxillary sinus augmentation.	-Thirteen patients; -One group DBBM with L-PRF, the other group DBBM; -Lateral window approach; -Two-stage implantation; -Twelve-month follow-up.	L-PRF in DBBM did not increase the amount of regenerated bone.
Powell et al., 2022 [40]	Case series	To investigate leukocyte- and platelet-rich fibrin (L-PRF) in sinus lift.	In the first case, L-PRF+ allograft was employed to support a maxillary hybrid denture by bilateral sinus augmentation. In the second patient, only L-PRF was used in the elevation of the Schneiderian membrane. In the third case, implant was placed after L-PRF/xenograft+ sinus augmentation, and a histology was provided six months later.	Dental implants were successfully placed in every patient. In the second case, freeze-dried bone allograft was used to offer around 4 mm of extra vertical height for implant insertion. Six months after sinus augmentation, histology from the third case showed that there was fresh, viable bone in contact with the xenograft.
Rapone et al., 2022 [3]	Retrospective study	To compare the long-term clinical results of bone regeneration treatments employing plant hydroxyapatite to demineralized anorganic bovine bone combined with PRP.	-Fifty-seven patients -Lateral wall protocol and split bone technique. -Two groups: Group Algipore^®^ (n = 29) Group Bio-Oss^®^ (n = 28); -Two-stage implant placement; -Seven-year follow-ups.	For both groups, this study revealed predictable outcomes over time.
Simonpieri et al., 2012 [9]	Case series with 6-year follow-ups	The lateral sinus lift procedure using only PRF clots and membranes with immediate implantation,	-Twenty-three lateral sinus elevations; -Classical lateral sinus-lift, Caldwell-Luc approach; -The Schneiderian membrane was covered with L-PRF membranes; -L-PRF clots to fill the subsinus cavity; -Follow-up was conducted at six months, a year, and then every year after that for 6 years.	The periimplant crestal bone height was consistent, and the level of the reconstructed sinus floor was always in continuity with the implant apical end.
Zhang et al., 2012 [41]	Prospective study with 6 months of follow-up	In this work, DBBM was used as a xenograft in conjunction with sinus augmentation to assess the effect of PRF on bone regeneration.	-Five maxillary sinus lifts treated with a mixture of Bio-Oss and PRF (study group); -Five maxillary sinus lifts treated only with Bio-Oss (control group).	After a six-month healing period, this study did not show either a benefit or a drawback of using PRF in conjunction with DBBM in maxillary sinus augmentation.

## Data Availability

Not applicable.

## Abbreviations

ALP	alkaline phosphatase
BMP	bone morphogenetic protein
DBBM	deproteinized bovine bone mineral
EGF	epidermal growth factor
FGF	Fibroblast growth factor
GBR	guided bone regeneration
HGF	hepatocyte growth factor
IGF	Insulin-like growth factor
L-PRF	leukocyte-platelet-rich fibrin
MBL	marginal bone loss
MC	mineralized collagen
MSFE	Maxillary sinus floor elevation
PCs	platelet concentrates
PDGF	Platelet-derived growth factor
PRF	Platelet-Rich Fibrin
PRP	Platelet-Rich Plasma
RBH	residual bone height before surgery
TBH	total bone height.
TGF	Transforming growth factor
VEGF	Vascular endothelial growth factor
